# Full-Thickness Rectal Biopsy in Children Suspected of Having Hirschsprung’s Disease: The Inconclusive Biopsy

**DOI:** 10.3390/children10101619

**Published:** 2023-09-28

**Authors:** Leise Elisabeth Hviid Korsager, Niels Bjørn, Mark Bremholm Ellebæk, Lene Gaardsmand Christensen, Niels Qvist

**Affiliations:** 1 Research Unit for Surgery, Odense University Hospital, University of Southern Denmark, 5230 Odense, Denmark; leiko@regsjaelland.dk (L.E.H.K.); niels.bjorn@rsyd.dk (N.B.); mark.ellebaek1@rsyd.dk (M.B.E.); 2 Centre of Excellence in Gastrointestinal Diseases and Malformations in Infancy and Childhood (GAIN), Odense University Hospital, 5000 Odense, Denmark; 3 Research Unit for Pathology, Odense University Hospital, University of Southern Denmark, 5230 Odense, Denmark; lene.gaardsmand.christensen@rsyd.dk

**Keywords:** Hirschsprung’s disease, full-thickness rectal biopsy, inconclusive biopsies, hematoxylin, eosin, immunohistochemistry

## Abstract

The diagnosis of Hirschsprung’s disease relies on histologically proven aganglionosis and nerve trunk hypertrophy in rectal biopsies. Although the frequency of inconclusive biopsies is relatively low, it is a relevant clinical problem. The aim of the present study was to investigate whether a re-evaluation of archived full-thickness biopsies (FTBs) stained with hematoxylin and eosin (HE), together with immune histochemical (IHC) staining, would be diagnostic in biopsies otherwise deemed inconclusive at initial examination with HE only. A total of 34 inconclusive biopsies in 31 patients were identified. From each tissue block, three slices were cut and stained with HE, S100 and calretinin. A blinded pathologist examined the tissue samples. At re-evaluation, one patient was found positive for HD and 11 negative for HD with both HE and IHC staining, respectively. In all 12 cases, the result was confirmed by the final diagnosis at a 5-year follow-up. The rest of the cases were deemed inconclusive. A re-evaluation of the remaining tissue from the biobank might have saved one third of the children from a re-biopsy. The value of adding IHC to conventional HE staining is dubious.

## 1. Introduction

Hirschsprung’s Disease (HD) is a congenital malformation characterized by the absence of ganglion cells in the submucosal myenteric plexus (Meissner) and the intramuscular plexus (Auerbach) in the bowel wall. Other histopathological features are hypertrophy of the submucosal nerve trunks, absent calretinin-immunoreactive mucosal innervation and increased cholinergic innervation. The pathology starts at the internal anal sphincter and extends proximally over various distances; it most commonly affects the rectum and sigmoid colon but may involve the entire colon and distal small intestine. The affected bowel is aperistaltic, causing fecal impaction above the affected bowel segment. The only effective treatment is surgical resection.

Diagnosis in children suspected of having HD ultimately relies upon a histopathological examination of a biopsy from the rectum 2–3 cm above the dentate line, obtained transanally either as an excisional full-thickness biopsy (FTB) or a suction biopsy (RSB). The main advantage of an FTB is the significantly larger tissue samples obtained for histological examination. The disadvantage is that it requires general anesthesia, unlike suction biopsies, which only need sedation. A lack of muscular tissue in an FTB will often result in a biopsy being deemed inconclusive by the pathologist with the recommendation of a re-biopsy, which may be bothersome for the child and parents. The reported frequency of inconclusive biopsies varies from 0 to 15% for FTBs [1,2].

Hematoxylin-and-eosin staining (HE) is the most common preparation and criterion standard used for histological examination to document the presence or absence of ganglion cells and nerve trunk hypertrophy [3]. Acetylcholinesterase enzyme histochemistry as a diagnostic adjunct was introduced decades ago [4], and later, immune histochemical staining (IHC) with antibodies against S100 antibodies [5] and calretinin [6,7,8] was introduced to improve diagnostic accuracy.

The aim of the present study was to investigate whether a re-evaluation of archived FTB biopsies stained with hematoxylin and eosin (HE), together with IHC staining, would be diagnostic in biopsies otherwise deemed inconclusive at initial examination with HE staining alone. The reference was the final diagnosis following repeated and sufficient FTBs, surgical specimens and a clinical follow-up after at least 5 years.

## 2. Material and Methods

During the period from January 2008 to December 2014 a total of 593 FTB rectal biopsies in 555 patients were performed. The median age was 20 months (range: 0–191) and the sample contained 322 boys and 233 girls. The patients were identified via the Danish National Pathology Data Base (Patobank^®^) and they had all been referred under suspicion of HD with severe constipation refractory to medical treatment and/or difficult and strenuous defecation without another obvious reason. Our clinical standard was to obtain a single full-thickness biopsy from the back of the rectum at least 2 cm above the dentate line. After formalin fixation, the biopsy was embedded in paraffin and 6–10 slices of 3–4 µm thickness were cut and stained with HE, and the remnant tissue was stored in the Patobank^®^. All the biopsies were performed by 4 experienced pediatric surgeons. 

**Figure 1:**
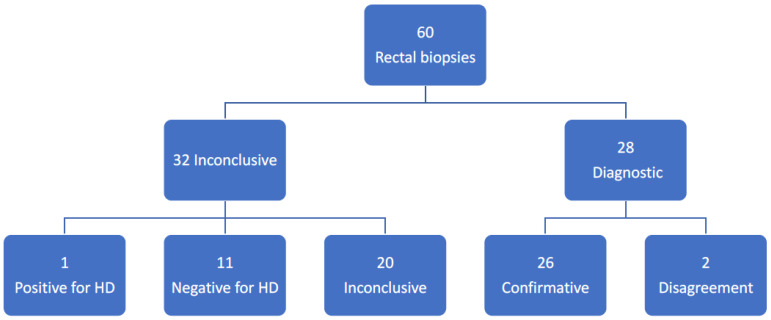
Results of the re-evaluation of the inconclusive and confirmative biopsies in 31 patients with primary inconclusive biopsies. HD: Hirschsprung’s disease.

In 31 patients, the original biopsies were deemed inconclusive at the initial histological examination by 3 experienced pathologists. There were 10 girls and 21 boys with a median age of 1 year and 3 months (range: 3 days to 14 years and 1 month). All children underwent a secondary biopsy session and 3 children a third because of an inconclusive biopsy at the re-biopsy. This left us with 34 eligible inconclusive biopsies for re-examination. The histological re-evaluation was performed by an experienced pathologist (LGC) who was not involved in the initial evaluation and blinded to clinical information and the previous results of the original histopathological examination. For quality control, the tissue from the final and sufficient FTBs (31 biopsies) were also re-examined Thus, a total of 65 archived formalin-fixed, and paraffin-embedded tissue blocks were eligible for analysis: 34 inconclusive and 31 diagnostic. Five biopsies were excluded from the analysis due to a lack of sufficient material for further examination, and thus, we ended up with 60 biopsies included in the study: 32 inconclusive and 28 diagnostic (Figure 1). Three 3–4 µm thick tissue slices were cut, and one was stained with HE and two with antibodies against S-100 (IN S100^®^, Agilent, Santa Clara, CA, USA) and calretinin (IE Calret^®^, Ventana Medical Systems, Marana, AZ, USA), respectively.

The results of the new histopathological examination were grouped into the following categories according to the HE and the IHC: inconclusive, positive for HD or negative for HD. For HE, a biopsy was defined as negative for Hirschsprung’s disease if there were one or more ganglion cells found in any layer, myenteric or submucosal; positive if no ganglion cells were identified; and inconclusive if there were non-specific findings. For IHC, a biopsy was defined as positive for HD if there was the visual presence of a positive reaction for S100 and no reaction for calretinin, and inconclusive if there were non-specific findings. In all patients, a follow-up (medical records) for at least 5 years was performed to confirm the final diagnosis.

This study was approved by the Danish Patient Safety Authority (3-3013-1110/1) and by the Danish Protection Agency (15/19986).

## 3. Results

The results are presented in Figure 1. Upon re-evaluation of the 32 inconclusive biopsies, one was found positive for HD and 11 negative for HD with both HE and IHC, respectively. In all 12 cases the result was confirmed by the final diagnosis at follow-up. Twenty cases were deemed inconclusive.

Following quality control of the 28 eligible diagnostic biopsies from the secondary and tertiary biopsies, a discrepancy between the original investigation and the re-evaluation was found in two patients. In one patent with confirmed HD, the re-evaluation correctly concluded aganglionosis at with HE but was inconclusive for IHC. In the other patient with no HD confirmed, the conclusion at re-evaluation was aganglionosis with HE but negative for HD with IHC. 

The reasons given by the pathologist to deem a biopsy inconclusive based on the original pathological report are presented in Table 1. A superficial biopsy was the most common.

## 4. Discussion

The results from the present study can be interpreted in several ways. A re-evaluation of the primary biopsies deemed inconclusive might have saved approximately one third of the children from another biopsy. The re-evaluation in these cases was in in concordance with the final diagnosis after at least a 5-year follow-up as a quality control measure for the re-evaluation. The results also showed that a re-evaluation of biobank tissue harbored the risk of both false-positive and false-negative diagnoses for HD. Finally, it was shown that the addition of IHC to HE staining had no significant influence on the results, but we cannot exclude that it may be helpful in some situations. 

It is generally accepted that the detection of a single ganglion cell via conventional HE either in the myenteric or submucosal plexus excludes the diagnosis of HD [9,10]. Some have recommended that a minimum of 50 HE levels must be examined to confirm aganglionosis [1]. Others have found that 10 HE-stained sections from different levels of the biopsy specimen would be enough to detect ganglion cells in most cases [11].

We cannot exclude that the investigation of more slices at the initial investigation could have reduced the number of inconclusive biopsies. A full-thickness biopsy may be challenging in especially small babies, but inconclusive biopsies were also seen in older children. Additional biopsies during the first session with a biopsy might have resulted in a lower frequency of inconclusive biopsies, but we chose to take only one biopsy to reduce the risk of complications. Only three slices were prepared for the re-evaluation, one for each staining method. This may, to some extent, explain why two thirds of the biopsies at the new re-evaluation of the primary biopsy were still classified as inconclusive. Our study has several limitations. The most important may be the low number of histological slices investigated and the retrospective design. The long-term clinical follow-up is a strength.

Nerve trunk hypertrophy is another criterion for the diagnosis of HD. Nerve trunk hypertrophy can be seen in HE at high magnification but may also be assessed via acetylcholinesterase staining or IHC. Although acetylcholinesterase staining has very high diagnostic sensitivity and specificity [4], the method has been abandoned by most laboratories due to complicated tissue handling with snap-freezing and has gradually been replaced by IHC. Several immune histochemical staining methods have been investigated and proposed for clinical use [12,13,14]. We chose to include calretinin and S100, which are the most investigated and best documented methods. An absence of calretinin staining confirms both the absence of ganglion cells and the presence of nerve trunk hypertrophy indicative of HD [8]. S100 reveals a prominent staining of the nerve trunks only, indicative of the nerve fiber proliferation typical of HD [5].

Although it has been shown that the addition of IHC to conventional HE may improve diagnostic sensitivity and specificity [15], this has, to the best of our knowledge, never been proven in randomized studies. In a study by Gonzalo et al., a retrospective analysis was conducted of 17 rectal suction biopsies that were deemed inconclusive at HE staining. Immunohistochemistry for calretinin was performed for all cases and showed that all patients with confirmed HD had no reaction to calretinin and that all without HD showed a reaction [8]. In a before-and-after study, the introduction of calretinin to conventional histology was investigated in a retrospective study including 82 patients undergoing suction biopsies for Hirschsprung’s disease. Before the introduction of calretinin immunochemistry, 37.8% of the biopsies were deemed inconclusive, and 11.9% were deemed inconclusive after. The difference was significant, but the authors concluded that the calretinin test may improve the diagnostic accuracy [16]. In another study on 131 biopsies, it was concluded that calretinin staining could overcome difficulties with dubious cases of HE and acetylcholinesterase staining [17]. Similar results have been reported for S100 [5]. Another retrospective study showed that calretinin coupled with S100 and PGP9.5 (protein gene product 9.5) immunostaining on suction rectal biopsies increased the sensitivity and specificity for diagnosing HD [13].

The use of neuron-specific enolase via the peroxidase–antiperoxidase technique is another immunohistochemical method. The perikarya of normal ganglion cells are strongly positive for NSE and are easily recognizable even when the nerve cells are immature. The hypertrophic submucosal axonal bundles characteristic of Hirschsprung’s disease is also readily demonstrable. In a retrospective study, HE and the neuron-specific enolase staining proved to be of equal value in the assessment of the presence of neurons in the rectal wall of patients with clinically suspected Hirschsprung’s disease [11].

Age at the time of biopsy, this may play a role in the sensitivity and specificity of different staining methods. In a retrospective study on calretinin, the percentage of inconclusive results was higher in patients younger than 1.5 months. In another retrospective study, RSB analysis identified HD in patients younger than 39 days old with only 50% sensitivity. In addition, RSB obtained from younger patients often led to inconclusive outcomes that required additional biopsies [18]. The patients with inconclusive biopsies in our study were represented by all ages.

In the choice between incisional or suction biopsies, the frequency of inadequate biopsies may be relevant. In a retrospective study with 133 suction biopsy procedures with 227 biopsies, inadequacy was found in 24.1% of the procedures compared to 0.9% in 125 incisional biopsy procedures with 140 biopsies [19].

Other modalities reported in the diagnosis of HD include contrast enema and anorectal manometry, alone or in combination with rectal biopsies. In a prospective study, 111 patients suspected of HD underwent all three tests. Children with positive results on two or more index tests or who continued to have significant bowel problems underwent an FTB as a reference. Clinical follow-up was the reference standard. RSB had the highest sensitivity (93%) and specificity (100%) rates. The values from colonic enema were 76% and 97% and those from anorectal manometry were 83% and 93%, respectively. Inconclusive test results occurred in 8 infants with colonic enema, in 15 infants with anorectal manometry and in 2 infants with RSB. The differences were not statistically different, and the authors concluded that RSB is the most accurate test for diagnosing HD and has the lowest rate of inconclusive test results [20].

The interpretation of HE as well as IHC rely on a subjective and visual evaluation of the presence or absence of ganglion cells and nerve trunk hypertrophy. Although the sensitivity and specificity for HD diagnosis for each separate staining method are very high (>95%) in most reports, there will always be a risk of false-negative and false-positive results for HD. The most serious is a false positive, which may result in an unnecessary surgical resection with the risk of devastating complications. In our control material, one patient was at risk of having a false-positive diagnosis if focus was placed upon the results of HE alone, but the supplementary IHC was negative for HD. In one patient with confirmed HD, the IHC was inconclusive. A false-negative result for HD is less serious. Therefore, the results of the histological examination of rectal biopsies for HD must be weighed against the clinical presentation. Any doubt should result in a re-biopsy. 

## 5. Conclusions

Full-thickness rectal biopsies for Hirschsprung’s disease may be deemed inconclusive by the pathologist for various reasons. A re-evaluation of the remaining tissue placed in the Patobank^®^ might have saved one third of the children assessed in this study from a re-biopsy. The value of adding IHC to conventional HE staining is dubious.

## Figures and Tables

**Table 1:** The reasons given by the pathologist to deem a biopsy inconclusive based on the original pathological report in 31 patients with 34 biopsies.

Reason	Number of Biopsies
Lack of tunica mucularis	23
Biopsy taken too close to the dentate line.	5
The Biopsy is too small	2
Others	4

## Data Availability

The data presented in this study are not publicly available due to data confidentiality.
